# Effects of antioxidant capacity-based dietary replacement of vitamin E with commercial products containing grape skin–green tea extracts or hydrolyzed wood polyphenols on poultry performance, metabolism, immune-related gene expression, and meat quality

**DOI:** 10.3389/fvets.2025.1608147

**Published:** 2025-07-07

**Authors:** M. Simoni, T. Danese, N. Mezzasalma, A. Goi, R. Pitino, G. Mantovani, M. Andrani, A. Costa, A. Plomaritou, L. Ragionieri, E. Tsiplakou, M. De Marchi, F. Righi

**Affiliations:** ^1^Department of Veterinary Science, University of Parma, Parma, Italy; ^2^Department of Agronomy, Food, Natural Resources, Animals and Environment (DAFNAE), University of Padova, Padova, Italy; ^3^Department of Veterinary Medical Sciences, University of Bologna, Bologna, Italy; ^4^Department of Animal Science, University of Thessaly, Larissa, Greece; ^5^Department of Animal Science, Agricultural University of Athens, Athens, Greece

**Keywords:** α-tocopherol, broiler, health status, organic production, plant feed additives

## Abstract

Natural antioxidants are widely investigated as potential substitutes for synthetic compounds suitable for use in organic diets. They are often included in diets based on literature and manufacturer recommendations, sometimes leading to inconsistent results. In this study, we replaced 0.05 g/kg of vitamin E in poultry diets (vitE) with commercial products containing grape skin and green tea extracts (GSGT) or hydrolyzed wood polyphenols (HWP), at doses calculated based on their *in vitro* antioxidant capacity measured by the 2.2′-azino-bis (3-ethylbenzothiazoline-6-sulfonic acid) test (ABTS). A total of 252 one-day-old male Ross 308 chicks, reared according to organic farming guidelines, were assigned to 9 pens in a randomized block design with 3 replicates per dietary treatment; 108 were slaughtered at 42 days as in the conventional production cycle, while the remaining were slaughtered at 84 days, in accordance with organic farming regulations. Overall, no differences were observed in performance, antioxidant capacity, hematological parameters, carcass and cut weights, meat quality, and survival rate among the dietary treatments. The control group had the highest plasma vitamin E levels (*p* < 0.0001), while at 42 days, the HWP diet increased plasma vitamin A (*p* = 0.041) and reduced ALT-GPT levels (*p* = 0.008). The GSGT diet reduced creatinine levels (*p* < 0.0001) and showed higher meat protein content compared to HWP (*p* = 0.024). Differences in gut histomorphology were observed at 42 and 84 days, with effects on specific intestinal regions. Natural antioxidants modulated immune-related gene expression, maintaining the innate immune system in an “alert” state compared to control. The GSGT and vitE groups showed the highest European Production Efficiency Factor (EPEF) at 42 days, while at 84 days, a higher EPEF was observed for the HWP and GSGT groups. In conclusion, HWP and GSGT can effectively replace vitamin E in poultry diets when balanced for antioxidant capacity, in both conventional and organic diets.

## 1 Introduction

To address recent European research priorities in organic farming, scientists have increasingly focused on investigating natural alternatives to synthetic vitamins in animal nutrition. Recent reviews have discussed the utilization of plant feed additives (PFA) as substitutes for vitamin E in animal diets (1–3). Due to their polyphenolic nature, PFAs, which are secondary metabolites of plants, have the potential to positively impact antioxidant status, biochemical parameters, welfare, growth performance, and tissue development of mammals and poultry (4, 5). However, negative effects due to high doses have also been reported, and the lack of standardized protocols for PFA application and usage has been highlighted (4, 5). Moreover, literature is lacking on the use of dietary natural alternatives to synthetic vitamins under organic farming conditions, particularly with slaughtering performed after 81 days of the rearing cycle (EU Regulation 2018/848).

Among the most promising and readily accessible PFAs are grape industry by-products, green tea extracts, and hydrolyzed polyphenols from wood (6–9). In line with circular production approaches, their use as feed supplements may contribute to the reduction of waste biomass from the agricultural sector (10).

Grape by-products have demonstrated positive effects on broiler and livestock health and performance, nutrient utilization, antioxidant status, intestinal microbiota, and carcass traits (6, 10). Grape pomace has also shown anthelmintic activity in sheep and immunostimulant properties in heat-stressed broilers (10), representing a sustainable and natural alternative to conventional feed supplements (10). Their availability is abundant, as 3.2 million hectares across the entire European continent are occupied by vineyard cultivations, with Italy, France, and Spain accounting for 75% of the total (11), and several by-products are generated during vinification. Among them, grape skin extract emerges as a valuable source of bioactive compounds (12). In fact, compared to grape seeds, grape skin contains a lower amount of proanthocyanidins but has a higher degree of polymerization and a higher content of dietary fiber and non-extractable polyphenols (10, 13).

Tea is the most widely consumed non-alcoholic beverage in the world, and the phytochemistry and toxicology of tea leaves are well-documented (14). In 2023, over 32 million tons of tea leaves were produced worldwide, representing a 141% increase in production compared to 10 years earlier (15). Green tea has bacteriostatic, anti-cancer, lipid metabolism regulatory, and immunomodulatory activity. Additionally, its polyphenols mitigate heat stress in poultry, enhancing antioxidant defense mechanisms, particularly through the cytoprotective molecular basis of epigallocatechin-3-gallate (EGCG), which protects against oxidative damage. On the other hand, its application in poultry and livestock is challenged by reduced palatability caused by the astringency of tannins (7). Additionally, several antinutritional and side effects have been reported, such as negative impacts on gut microbiota, meat quality, shear force, feed intake, and efficiency (16). High EGCG doses can inhibit basolateral iron efflux from erythrocytes through non-transportable iron–polyphenol complexes, reducing intestinal iron absorption and affecting liver metabolism (17, 18).

Wood residues globally account for 0.25 km^3^ per year (15). Among them, wood waste extracts represent a valuable bio-functional source of phenolic acids, including ferulic, caffeic, protocatechuic, gallic, *p*-coumaric, and chlorogenic acids. All these extracts exhibit strong free radical scavenging properties and antimicrobial activities (19) and seem to have the potential to substitute synthetic vitamin E, either partially or entirely, when administered at specific doses in poultry diets (2, 3, 20). On the other hand, these antimicrobial effects may negatively impact gut microbiota (21).

From the analysis of the available literature on the topic, it appears that only doses selected based on literature data or manufacturer recommendations were tested, without performing any real antioxidant capacity evaluation on the product to be tested, aimed at balancing the experimental and control diets for this property (2). Because of this lack of standardization, along with the wide variability in the composition of the PFAs (22), the results of studies on natural antioxidants are often inconsistent and difficult to interpret. Assuming that, regardless of their formula or composition, PFAs can be characterized by their antioxidant capacity through specific laboratory analysis (e.g., ABTS test, expressing the antioxidant activity as vitamin E equivalents), we hypothesize that natural antioxidants can completely replace vitamin E in chicken diets, provided that they are dosed based on their antioxidant capacity to obtain diets balanced for this characteristic.

Thus, the objective of the present trial was to assess the effect of replacing synthetic vitamin E with two commercial products containing grape skin and green tea extracts or hydrolyzed wood polyphenols, based on their measured antioxidant properties, on poultry health, oxidative status, efficiency, performance, metabolism, immune-related gene expression, and meat and carcass quality, while maintaining an equivalent dietary antioxidant capacity. Considering their potential to replace vitamin E in organic diets, this study was conducted simulating organic farming practices, evaluating the effects on animals reared up to 84 days, in accordance with organic farming regulations.

## 2 Materials and methods

The poultry rearing and handling procedures were approved by the ethical committee for the care and use of experimental animals of the University of Parma, Italy (PROT. N.15/CESA/2021) and were conducted in accordance with Italian law (Decreto legislativo no. 26/2014) and the EU Directive 2010/63/EU on the protection of animals used for scientific purposes. The trial was performed in approved animal stables of the Department of Veterinary Science of the University of Parma (Italy).

### 2.1 Plant feed additives tested, antioxidant capacity measurement, and diet antioxidant capacity adjustment

The PFA tested were two commercial products, Raitee (BIOTRADE di Malavasi Claudio & C. S.n.c., Mirandola, Modena, Italy), consisting of a mixture of grape skin (Raisinox^®^ 80Q, SARB 1952 S.r.l., Lecce, Italy) and green tea (Athesis di Turazza Marco, Verona, Italy) extracts at a 2:1 ratio (GSGT; blended to exploit the synergistic activity of the two ingredients and limit the impact of their antinutritional factors), and Oxistim (G. A. B. s.r.l, Verona, Italy), a complex of hydrolyzed polyphenols from wood (HWP). The evaluation of the antioxidant capacity of these products was performed using a 2.2′-azinobis (3-ethylbenzothiazoline-6-sulfonic acid) radical cation (ABTS) assay, with minor modifications (23). The ABTS test provided results as Trolox (a water-soluble vitamin E analogue) equivalents that were directly employed to calculate the PFAs’ doses capable of matching the dietary antioxidant capacity obtained using 0.1 g/kg of vitamin E, which represented the control treatment. To further characterize the products studied, antioxidant activity tests were also performed using Folin and 2,2-diphenyl-1-picrylhydrazyl (DPPH) methods. The results are reported in Table 1.

**Table 1:** Antioxidant capacity (mean ± SD) of the natural antioxidants grape skin and green tea extract (GSGT), and hydrolyzed wood polyphenols (HWP).

Commercial products	FOLIN, mg/g	ABTS, μmol Trolox/g	DPPH, μmol Trolox/g
GSGT^*^	140.21 ± 8.95	155.56 ± 6.25	163.58 ± 13.61
HWP	21.32 ± 1.60	31.82 ± 1.76	31.35 ± 2.78

ABTS, 2.2′-azino-bis (3-ethylbenzothiazoline-6-sulfonic acid); DPPH, 2,20 -diphenyl-1-picrylhydrazyl. *Calculated as a weighted average of grape skin (GS) and green tea (GT) extracts; the two extracts were mixed at a ratio of 2:1 blended to exploit the synergistic activity of the two ingredients and limit the impact of their antinutritional factors. The GS and GT individually were characterized by the following antioxidant capacities: 147.36 ± 2.87 and 125.91 ± 21.11 mg/g of FOLIN; 133.84 ± 4.24 and 199.00 ± 10.29 μmol Trolox/g of ABTS; 159.98 ± 8.80 and 170.79 ± 23.24 μmol Trolox/g of DPPH.

### 2.2 Animals and housing

A total of 252 one-day-old Ross 308 male broiler chicks (weighing 41.75 ± 3.05 g) were purchased from a commercial hatchery (Aglietto natura S.r.l., Vercelli, Italy). This breed was selected because it is the most commonly used in conventional farms and, as reported by (24), due to economic reasons, it is often adopted in organic production even if they are less resistant to diseases. The chicks were randomly allotted among 9 indoor floor pens (28 birds per pen) in a randomized block design with 3 replicates per treatment; for each treatment, the replicates were distributed at the extremities and in the middle of the shed. Chickens received vaccinations on their first day of life against Newcastle disease (Hatchpak Avinew, lot n° 197 U81, Boehringer Ingelheim International GmbH, France), infectious bronchitis (Hatchpak IB H120, lot n° 207Z11, Boehringer Ingelheim International GmbH, France), and Marek’s disease virus strain CVI988 (Nobilis® Rismavac, lot n° A1239A, MSD Animal Health, Merck & Co., Inc., Rahway, NJ, USA). The pens were uniform in size, and their surface area was set to comply with the maximum density recommendation for organic rearing, considering the final expected weight of the broilers, at 21 kg/m^2^ (25). The birds were reared in a controlled environment room where temperature and humidity were monitored twice daily (morning and afternoon) using a thermo-hygrometer (Karl Koch GmbH, Külsheim, Germany) in three positions within the shed. During the growth phase, each pen was equipped with a 250 W UV bulb light to maintain an average temperature of 40°C during the first week. In the following period, the temperature was gradually reduced by 4°C per week until reaching an average value of 24°C on day 28, which was maintained throughout the experimental period. Environmental humidity averaged 61.5% during the entire trial. Throughout the experiment, a 16-h light interval was provided daily. Animals in each pen had *ad libitum* access to both water and feed. The water was supplied by automatic bell-shaped drinkers cleaned weekly, while feed was dispensed using plastic feeders. Both drinkers and feeders were replenished and checked twice daily. The animals were reared under organic-like conditions, and the diet formulated did not include synthetic amino acids, prebiotics, or other technological additives. Moreover, no substances banned from organic farming, such as antibiotics and pesticides commonly employed in conventional systems, were used.

At 42 days of age, a total of 108 broilers (12 per pen) were sent to the slaughterhouse (Le Castellane S.c.a., Castelvetro di Modena, Italy) after a 12-h fasting period, according to the breed guidelines recommendations (26), to compare the performance parameters with those of a conventional rearing cycle; the remaining were sent to the same slaughterhouse on the morning of the 84th day of age, in compliance with the organic livestock farming guidelines (EU Regulation 2018/848). Animals were slaughtered after a 12-h fasting period, according to the breed guidelines recommendations (26), through electrical stunning followed by bleeding in accordance with Council Regulation (EC) No. 1099/2009 on the protection of animals at the time of killing (27). Blood samples were collected after stunning, during the bleeding phase.

### 2.3 Dietary treatments and feeding practices

The feedstuff formulations across the three dietary treatments (**DT**) were similar, designed to be isocaloric, isonitrogenous, and to possess identical antioxidant capacity regardless of the type of antioxidant employed. Basal diets, including starter (days 0–21), grower (days 22–42), and finisher (days 43–84) feedstuffs, were commercial formulations provided by a specialized industry (Fanin S.p.a, Vicenza, Italy). These feedstuffs were supplemented with 5 g/kg of macro- and microminerals and vitamin premix (based on the ICV3570 premix formula, ITACA EUROPA S. A, Barcelona, Spain), excluding vitamin E, and provided *ad libitum* throughout the entire experimental period. All diets were formulated to meet or exceed the minimum nutrient requirements for broiler chickens of the National Research Council. The DT were a control diet, vitE (basal diet + 0.05 g/kg of vitamin E, as suggested in the breed guidelines and kept constant during the whole trial); a grape skin–green tea diet, GSGT (basal diet + 0.746 g/kg of a 2:1 blend of Raisinox^®^ 80Q and green tea); and a hydrolyzed polyphenols diet, HWP(basal diet + 3.65 g/kg of Oxistim). From each batch of feedstuff preparation (2 batches per phase per DT), a 1 kg sample of the diet was collected and chemically analyzed as described in the literature (28). The composition of the diets provided to the animals is presented in Table 2. The apparent metabolic energy was calculated from the diet nutrient composition using the equation reported in the literature (29).

**Table 2:** Ingredients and chemical composition (expressed as mean ± SD) of the basal diets fed during the beginning (1–21 d of age), growing (22–42 d of age), and finishing period (43–84 d of age).

Items	Starter	Grower	Finisher
	1–21 d	22–42 d	43–84 d
Ingredients, % DM			
Soybean cake^a^	40.6	29.0	10.7
Corn	36.9	19.8	18.8
Wheat meal	8.14	15.7	18.8
Roasted soybean^b^	8.02	18.9	14.9
Barley meal	0.97	11.7	32.0
Soybean oil	1.36	1.20	1.38
Defluorinated monocalcium phosphate	1.65	0.87	1.87
Calcium carbonate	1.47	0.79	0.98
Sodium bicarbonate	-	0.79	0.09
Sodium chloride	0.96	0.79	0.09
Mineral and vitamin premix^c^	0.50	0.50	0.50
Chemical composition, %DM			
DM, % as fed	89.9 ± 0.31	91.2 ± 0.21	90.7 ± 2.11
Ash	8.08 ± 0.13	6.79 ± 0.34	6.47 ± 1.00
CP	24.5 ± 0.27	24.0 ± 3.48	18.3 ± 0.07
Crude fiber	4.44 ± 0.78	4.39 ± 0.86	4.88 ± 1.58
Ether extract	6.76 ± 0.27	7.36 ± 0.29	6.73 ± 0.26
aNDF	12.9 ± 0.35	13.4 ± 0.74	13.9 ± 2.11
ADF	4.64 ± 0.49	4.41 ± 0.49	5.46 ± 1.47
Lignin	1.89 ± 1.29	1.49 ± 1.17	2.25 ± 0.79
Starch	34.3 ± 1.51	30.7 ± 1.50	33.8 ± 1.86
NFC^d^	47.7 ± 0.34	48.3 ± 3.33	54.7 ± 2.82
AME^e^ (kcal/kg DM)	5,816 ± 55.9	5,369 ± 58.7	5,366 ± 32.55

a Mechanically defatted soybean meal.

b Full-fat soybean meal, containing soybean hulls.

c Mineral–vitamin premix manufactured by ITACA EUROPA S. A. (Barcelona, Spain) excluding vitamin E, provides vitamin A 12,500 UI, vitamin D3 5,000 IU, vitamin K3 1.65 mg, vitamin B1 1.65 mg, vitamin B2 5 mg, vitamin B6 1.65 mg, vitamin B12 0.03 mg, niacin 33.3 mg, biotin 0.33 mg, choline 667 mg, folic acid 0.43 mg, manganese 60 mg, zinc 100 mg, iron 67 mg, copper 17 mg, iodine 1.0 mg, and selenium 0.33 mg per 1 kg of diet.

d NFC, non-fibrous carbohydrate.

e AME, apparent metabolic energy was calculated based on the equation reported by Noblet et al. (29).

### 2.4 Measurements and sampling

Feed and water consumption (FC and WC, respectively) were measured daily on a pen basis using a gravimetric method, and concurrently, health status and mortality were evaluated and recorded. Dead animals were removed daily, and their weight was recorded.

Individual body weight (BW) was measured at placement and repeated weekly until 42 days, and then every 2 weeks until 84 days of age.

At the slaughterhouse, two individual plasma samples were collected during bleeding in 9 mL lithium heparin vacuum tubes (Ref: 455084, Vacuette® tube LH Lithium Heparin, Greiner Bio-One GmbH, Kremsmünster, Austria) for vitamin E and metabolic profile determinations. Additionally, one individual whole blood sample was collected in 9 mL EDTA vacuum tubes (Ref: 455036, Vacuette® tube K3E K3EDTA, Greiner Bio-One GmbH, Kremsmünster, Austria) for complete blood count (CBC). For the antioxidant activity determination, one plasma sample was collected in 9 mL EDTA vacuum tubes from 6 slaughtered animals randomly selected per pen. Among these, from 3 broilers per pen, 2 additional whole blood samples were collected for plasmatic vitamin A (EDTA vacuum tubes) and immune-related gene expression analysis through peripheral blood mononuclear cells (PBMC) isolation (lithium heparin vacuum tubes).

After slaughtering, the entire coelomic content, including proventriculus, gizzard, duodenum–pancreas, jejunum, ileum, cecum, colon, heart, spleen, kidneys, liver, bursa of Fabricius, testicles, and the eviscerated carcass, were weighed. Furthermore, 2 cm long samples from the duodenum (at the level of the duodenal loop), jejunum (at the midpoint between the end of the duodenal loop) and the position of Meckel’s diverticulum (30), ileum, and colon taken approximately 2 cm before and after the ileocecal–colonic junction, respectively, according to the literature (31) were collected and immediately fixed in 10% neutral buffered formalin for histological analysis.

Eviscerated carcasses were collected and analyzed at the food laboratory of the Department of Agronomy, Food, Natural Resources, Animals and Environment of the University of Padova (Legnaro, Italy). Legs, wings, and breasts were separated from the carcasses and individually weighed. Major pectoralis muscles were excised to measure pH, color, and were prepared for the determination of meat quality.

### 2.5 Samples analysis

#### 2.5.1 Blood analysis

Plasma samples were obtained by centrifuging the whole blood at 3000 x *g* for 15 min at 4°C and were preserved at −20°C for metabolic profile analysis and at −80°C for vitamins and total antioxidant capacity determination and PBMC isolation.

The plasmatic α-tocopherol concentration was determined as described in the literature (32), using an iCheck fluorometer–spectrophotometer (iCheck Vitamin E; BioAnalyt GmbH, Teltow, Germany). The plasmatic content of vitamin A was assessed through a Chicken Vitamin A kit assay (ELISA kit) according to the manufacturer’s instructions (Assay Genie, Dublin).

The metabolic profile was analyzed at the Clinical Biochemistry Laboratory of the Istituto Zooprofilattico Sperimentale della Lombardia e dell’Emilia Romagna “Bruno Ubertini” (IZSLER, Brescia, Italy) using an ILab 650 automatic multiparametric analyzer for clinical biochemistry (Werfen Instrumental Laboratory S.p. A., Italy) with the related reagents. A colorimetric assay was used for all tested parameters, except for: (i) total cholesterol and high-density lipoproteins (HDL), which were tested with a dichromatic colorimetric end-point assay (33); (ii) alanine aminotransferase (ALT) and creatine kinase (CK), tested through an enzymatic kinetic colorimetric assay; (iii) gamma-glutamyl transpeptidase (GGT), tested by the enzymatic kinetics Szasz/Persijn method (IFCC, International Federation of Clinical Chemistry and Laboratory Medicine); (iv) urea, which was tested by the urease method. Non-esterified fatty acids (NEFA) and *β*-hydroxybutyric acid (BHBA) were analyzed by the same instrument using a colorimetric method with the “NEFA” and “RANBUT” kits (Randox Laboratories Ltd., Ireland).

The analysis of blood parameters was performed using an automated Cell Dyn 3,500 Plus hematology analyzer (Abbott Diagnostics, Lake Forest, IL, USA). These included measurements of red blood cell count (RBC), hemoglobin (HB), hematocrit (HCT), mean corpuscular volume (MCV), mean cell hemoglobin (MCH), mean cell hemoglobin concentration (MCHC), red cell distribution width (RDW), and platelets (PLT). Additionally, monocytes (MONO), lymphocytes (LYM), neutrophils (NEU), eosinophils (EOS), and basophils (BAS) were also assessed.

The total antioxidant capacity (FRAP: ferric reducing ability power; ABTS: 2,2′-azino-bis (3-ethylbenzothiazoline-6-sulfonic acid)) and malondialdehyde (MDA) concentration were determined spectrophotometrically (GENESYS 180, Thermo Fisher Scientific, Massachusetts, USA) in plasma, aiming to assess the oxidative status of the animals (32, 34).

#### 2.5.2 Histological analysis

Following fixation, intestinal samples were rinsed with water and dehydrated in a graded ethanol series. They were then clarified in xylene and embedded in paraffin. Cross-sections, 5 μm thick, were cut with a rotary microtome (Slee Cut 6,062, Slee Medical, Mainz, Germany). The sections were stained with hematoxylin–eosin (H&E). Whole slide images (WSI) of histological slides were obtained using a digital slide scanner (Nanozoomer S-60, Hamamatsu, Japan) for histomorphometric analysis.

The digital image processing software NDP view 2.6.13 (Hamamatsu, Japan) was used to measure the following morphometric parameters: tunica mucosa thickness (TMT), villus height (VH), crypt depth (CD), lamina muscularis mucosae thickness (MMT), and the thickness of the inner circular (Circ M) and outer longitudinal (Long M) layers of the tunica muscularis. The TMT was measured as the distance from the apex of the villus to the lamina muscularis mucosae. The VH was measured as the distance from the apex of the villus to the junction of the villus and crypt (35). The CD was measured from the base of the villus to the lamina muscularis mucosae (36). Five villi per sample were measured, and data were expressed in μm; only vertically oriented villi and crypts were measured. No attempt to correct for possible over- or under-estimation was made during image processing, ensuring that measurements were taken under identical and blinded conditions.

#### 2.5.3 Meat quality analyses

The pH of the meat was assayed using a model HD2107.2 Delta Ohm (Delta Ohm, Padova, Italy) with a high-precision pH meter (± 0.002 pH units). A colorimeter (CM-600d, Konica-Minolta Sensing Inc., Ramsey, NJ) was used to evaluate the meat color; the measurement was performed on the exterior surface of intact skinless breast muscle. The meat color was expressed as lightness (L∗), redness (a∗), and yellowness (b∗) color indices (CIE, 1976). To obtain a single value for each sample, three measurements were performed at different points on the muscle’s surface and averaged.

A muscle slice (1 × 7 × 3 cm) of the breast was cut, weighed, vacuum-packed, and cooked in a water bath at 80° C for 1 h. The sample was removed from the bag, and the drip was drained. After cooling, the cooked mass was weighed to determine weight loss. Shear force (N/g of cooked meat) was measured using the same slice with an LS5 Single Column Bench Mounted (AMETEK Lloyd Instruments Ltd., West Sussex, UK) equipped with an Allo-Kramer shear compression cell with 10 blades (8 × 7 cm) 22 mm thick, using a 500-kg load cell with a cutting speed of 500 mm/min. Data were analyzed using NEXYGEN PLUS 3 software (AMETEK Lloyd Instruments Ltd., West Sussex, UK).

The leftover portion of each raw breast sample was ground and divided into two aliquots. The first aliquot of 150 g was used to collect the NIR spectra, and the second aliquot of 50 g was stored at −20° C for fatty acid (FA) content determination.

Chemical parameters (i.e., moisture, fat, and protein contents) were assayed on 100-g samples analyzed using the NIR spectrometer FoodScan (FOSS, Hillerød, Denmark), with calibrations previously validated (37) against officially approved chemical methods such as AOAC Official Methods for fat (960.39) and protein (992.15). Visible/NIRS analysis was performed with NIRS DS2500 (FOSS, Electric A/S, Hillerød, Denmark). Thirty grams of ground meat were placed in a FOSS large glass cup (diameter 105 mm, depth 35 mm) and scanned from 400 to 2,500 nm wavelength with a 0.5 nm increment. The spectrum of each sample was obtained as an average of 32 sub-spectra collected at different points during the automatic rotation of the cup, using Mosaic software (FOSS, Hillerød, Denmark), and converted to absorbance [log(1/reflectance)] to develop the prediction models. The same amount of sample was then transferred to a glass-bottom sample cup (diameter 140 mm, depth 14 mm) and scanned by FoodScan (FOSS Electric A/S, Hillerød, Denmark) from 850 to 1,050 nm every 2 nm. This instrument recorded 16 sub-spectra and averaged them to obtain a single spectrum for each sample.

The meat content of vitamin A was assessed through a Chicken vitamin A kit assay (ELISA kit) according to the manufacturer’s instructions (Assay Genie, Dublin). Briefly, each sample was homogenized in 20 mL of 1x PBS (including protease inhibitors) and stored overnight at −20°C. To achieve a complete breakdown of cell membranes, the samples were subjected to two freeze–thaw cycles and further sonicated. Finally, the samples were centrifuged for 5 min at 5000 × *g*. The supernatant was collected and immediately assayed.

#### 2.5.4 Immune-related gene expression analysis

##### 2.5.4.1 Isolation of PBMC

Chicken PBMC (a heterogeneous group of round-nucleated immune cells, mainly comprising T, B, and NK lymphocytes, as well as monocytes) were isolated from 3 to 4 mL of chicken blood samples collected in lithium–heparin tubes. The blood samples were layered on Histopaque-1077^®^ solution (1:1, v/v, Sigma, St. Louis, MO) and spun at 400 × *g* for 30 min. Mononuclear cells were collected from the gradient interface, and the plasma suspension was combined and washed three times with antibiotic-free RPMI 1640 (Gibco, Carlsbad, CA, USA). The concentration was then adjusted to 2 × 10^6^ cells/mL using Trypan blue (Sigma) with a TC20™ Automated Cell Counter (BioRad, Hercules, CA, USA).

##### 2.5.4.2 RNA extraction and reverse transcription

Total RNA was isolated from chicken PBMC using a EuroGold Trifast™ kit solution (Euro-clone, Milan, Italy) according to the manufacturer’s instructions and reverse-transcribed to generate complementary DNA (cDNA) using oligo-dT primers (Bioneer; Daejeon, Korea). Purity (260/280 nm ratio) and concentration (at 260 nm) were assessed using a BioSpectrometer® (Eppendorf AG, Hamburg, Germany). The RNA samples were treated with DNase (Merck), and 1 μg/20 μL was reverse-transcribed using HiScript® III GSGT SuperMix (Vazyme Biotech Co.; Nanjing, China). The reverse transcription was performed using a StepOne™ thermocycler (Applied Biosystems, StepOne™ software v.2.3) according to the manufacturer’s instructions under the following thermal conditions: 2 min at 45°C, 15 min at 37°C, followed by 5 s at 85°C. The cDNA samples were stored at − 20°C.

##### 2.5.4.3 Real-time quantitative PCR (qPCR)

The cDNA samples were used as templates for qPCR, which was performed using a StepOne™ thermocycler (Applied Biosystems, StepOne™ software v.2.3). The cDNA (20 ng/20 μL) was amplified in duplicate using Fast PowerUp™ SYBR™ Green Master Mix (Applied Biosystems; Foster City, CA, USA) and specific primer sets (Eurofins Genomics, Ebersberg, Germany). Details of each primer set are presented in Supplementary Table 1. Samples were maintained at 95°C for 20 s and then subjected to 40 cycles consisting of a denaturation step at 95°C for 3 s, followed by an annealing/extension step at 60°C for 30 s. Data were analyzed using the 2 − ΔΔCt method, in which the expression levels of each gene were normalized to the reference gene *β*-actin (ACTB) (38, 39). The reference βAct gene was selected among other tested reference genes (i.e., HPRT1, GAPDH) as the endogenous control according to minimal intra−/inter-assay variation. A melting curve analysis for the specific amplification control was performed (60–95°C) at the end of the amplification cycles. No-reverse transcription controls and no-template controls (NTC) were included, and the latter considered negative and reliable if Ct was ≥ 35.

### 2.6 Calculations

Feed and water conversion ratios (FCR; WCR) were calculated weekly and at the end of each life cycle by pen, by calculating the cumulative FC or WC, respectively, divided by the total BW (including the weight of dead animals) determined at the considered interval. Water efficiency (WE) was calculated at slaughter by pen, dividing the total BW per pen by cumulative WC at the considered interval. The water-to-feed ratio (W: F) was calculated weekly as a ratio between the cumulative WC and FC per pen in the considered interval. The weekly BW gain (BWG) was calculated by pen as the difference between the mean BW of two consecutive weeks. Survival rate was monitored throughout the experiment and expressed as a percentage of living birds in each group at the end of each period (0–42 and 43–84 d). The estimated cumulative (EC) survival rate (0–84 d) was calculated considering the proportion of animals that died in the first period, as reported in the following equations.


$$\text{Survival rate}\%(0-42\;d)=\frac{\text{Live animals}\;\mathrm{at}\;d\;42}{\text{Animals allocated}\;\mathrm{at}\;d\;0}*100$$

$$\begin{array}{l} \text{Survival rate}\%(43-84\;d)=\\ \frac{\text{Live animals}\;\mathrm{at}\;84\;d}{\text{Remaining animals}\;\mathrm{at}\;d\;43\;(\text{after slaughter})}*100 \end{array}$$

$$\begin{array}{l} \mathrm{EC}\;\text{survival rate}\%(0-84\;d)=\\ \frac{\left(\text{Live animals}\;\mathrm{at}\;42\;d*\text{Survival rate}\%43-84\;d\;\right)}{\text{Animals allocated}\;\mathrm{at}\;d\;0}*100 \end{array}$$

The VH-to-CD ratio (VH/CD) was calculated individually at the two slaughtering intervals. The European Production Efficiency Factor (EPEF), a parameter used in the poultry industry to evaluate the performance and overall efficiency of poultry production systems, was calculated with the following formula:


$$\text{EPEF}=\mathrm{ADG}*\frac{\text{Survival rate}\;(\%)}{\mathrm{FCR}}*10$$

### 2.7 Statistical analysis

The data were analyzed using the software SPSS (IBM SPSS Statistics for Windows, Version 29.1; IBM Corp., Armonk, NY). Prior to performing the statistical analysis, normal distribution of the data was confirmed using the Kolmogorov–Smirnov and Shapiro–Wilk normality tests. The general linear model (GLM) was used to assess significant effects of DT. The Bonferroni test was performed as a *post hoc* evaluation. Measurements were performed at different levels:

- Individual bird: when one value per animal at each time point was available. Phenotypes included BW, organ weight, carcass weight, hematological parameters, and metabolic profile at slaughter. In a representative subsample of animals, the plasmatic content of vitamins, antioxidant capacity, histological evaluations, meat quality parameters, and immune-related gene expression were also determined. The DT was included in the model as a fixed factor, and the pen was considered as a random effect along with the residual.
- Pen: where one average value per group of birds was available. Phenotypes included animal-based data (FC, WC, BWG, FCR, W: F, WE, WCR) at different time points.

The DT was included in the model as a fixed factor, and the random factor was the residual. The Bonferroni test was performed as a *post hoc* evaluation. Finally, the survival rate—expressed as the number of living animals per group per period—was calculated for each experimental group and compared using the Chi-squared test. For all the analyses carried out, the level of significance was set at 0.05, while the trend threshold was set at *p* ≤ 0.1.

## 3 Results

### 3.1 Growth performance and feed and water consumption

The FC, WC, BWG, FCR, WCR, and W: F were similar across the DT (Table 3, Supplementary Table 2) with the exception of W: F, which was higher in the HWP group compared to the GSGT group at 42 days of age (2.46 vs. 2.67, respectively; *p* = 0.042), as outlined in Supplementary Table 2.

**Table 3:** Growth performance, feed and water consumptions of broilers fed with different dietary treatments (vitE, control diet; GSGT, grape skin–green tea diet; HWP, hydrolyzed wood polyphenols diet).

Variables	Dietary treatments			SEM	*p*-value
	vitE	GSGT	HWY		
BW day 0, g	41.9	41.8	41.5	0.19	0.620
0 to 21 d of age					
FC, g	1,130	1,131	1,166	13.1	0.497
WC, L	3.09	2.97	3.15	0.093	0.568
BWG, g	831	806	845	8.40	0.280
FCR	1.30	1.33	1.33	0.012	0.444
W: F	2.73	2.56	2.71	0.092	0.751
WCR	3.53	3.40	3.60	0.100	0.751
22 to 42 d of age					
FC, g	3294.18	3318.21	3,232	35.6	0.660
WC, L	8.58	8.27	9.04	0.200	0.329
BWG, g	1,566	1,643	1,560	26.5	0.418
FCR	1.96	1.98	2.03	0.029	0.644
W: F	2.52	2.42	2.65	0.047	0.145
WCR	4.94	4.59	5.20	0.136	0.205
43 to 84 d of age					
FC, g	10,300	10,119	10,050	335.49	0.963
WC, L	49.7	46.71	45.2	3.27	0.879
BWG, g	3,897	4,105	4,161	138.3	0.768
FCR	3.26	2.36	2.85	0.250	0.386
W: F	4.80	4.86	4.77	0.361	0.996
WCR	14.80	12.5	12.8	1.15	0.731

FC, feed consumption; WC, water consumption; BWG, body weight gain; FCR, feed conversion ratio; W: F, water-to-feed ratio; WCR, water conversion ratio.

### 3.2 Antioxidants traits, blood cell count, and metabolic profile

Plasmatic vitamin content and antioxidant capacity are reported in Table 4. As expected, the vitE group showed the highest plasmatic α-tocopherol content at 42 d (7.30 vs. 5.00 vs. 5.10 mg/L, respectively, for vitE, GSGT, and HWP; *p* < 0.0001) and, consistent with this result, also at 84 d of age (4.70 vs. 3.40 vs. 3.40 mg/L for vitE, GSGT and HWP; *p* < 0.0001). However, the HWP group showed the highest plasmatic content of vitamin A at 42 days (2.93 mg/L; *p* = 0.041), while no differences were found in the oxidative status parameters considered among the DT.

**Table 4:** Plasmatic vitamin content and antioxidant capacity of broilers fed with different dietary treatments (vitE, control diet; GSGT, grape skin–green tea diet; HWP, hydrolyzed wood polyphenols diet).

Variables	Dietary treatments			SEM	*p*-value
	vitE	GSGT	HWP		
42 d					
Plasmatic vitamin content					
Vit. A, ng/ml	1.85^b^	1.87^b^	2.93^a^	0.209	0.041
Tocopherol, mg/L	7.30^a^	5.00^b^	5.10^b^	0.156	<0.0001
Antioxidant capacity					
FRAP	3.70	3.50	3.60	0.091	0.831
ABTS	22.7	23.1	22.6	0.26	0.768
MDA	0.829	0.776	0.911	0.036	0.329
84 d					
Plasmatic vitamin content					
Vit. A, ng/ml	2.60	2.40	3.50	0.602	0.741
Tocopherol, mg/L	4.70^a^	3.40^b^	3.40^b^	0.110	<0.0001
Antioxidant capacity					
FRAP	4.30	4.30	4.40	0.150	0.966
ABTS	23.5	22.9	22.3	0.354	0.397
MDA	0.843	0.884	0.956	0.033	0.384

FRAP, ferric reducing ability power; ABTS, 2.2′-azino-bis (3-ethylbenzothiazoline-6-sulfonic acid); MDA, malondialdehyde. ^a,b^ Groups not bearing the common superscript are significantly different.

The blood cell count results showed no differences among the DT for any of the parameters considered at both 42 and 84 d of age (Table 5).

**Table 5:** Blood count cells of broilers fed with different dietary treatments (vitE, control diet; GSGT, grape skin–green tea diet; HWP, hydrolyzed wood polyphenols diet).

Variables	Dietary treatments			SEM	*p*-value
	vitE	GSGT	HWP		
42 d					
RBC, M/μL	1.84	2.74	1.73	0.314	0.354
HB, g/dl	6.67	6.42	6.49	0.353	0.969
HCT, %	22.9	22.9	21.3	1.26	0.909
MCV, fL	125.6	125.7	125.4	0.756	0.774
MCH, pg	36.8	36.9	40.0	1.01	0.322
MCHC, g/dL	29.5	29.3	31.2	0.645	0.240
RDW, %	8.62	8.44	8.44	0.167	0.845
PLT, K/μL	12.11	4.55	6.38	2.629	0.493
MONO, %	1.22	1.11	1.00	0.273	0.963
LYM, %	32.7	37.7	40.0	1.62	0.274
NEU, %	63.4	57.9	56.4	1.62	0.228
EOS, %	2.44	3.22	1.94	0.514	0.469
BAS, %	0.111	0.111	1.61	0.361	0.158
84 d					
RBC, M/μL	2.59	2.45	2.39	0.041	0.169
HB, g/dl	8.52	7.95	7.76	0.119	0.120
HCT, %	31.0	29.4	28.5	0.423	0.069
MCV, fL	120.0	119.8	120.2	0.603	0.993
MCH, pg	33.23	32.80	32.99	0.493	0.908
MCHC, g/dL	27.62	27.28	27.33	0.316	0.847
RDW, %	8.60	8.51	9.05	0.178	0.405
PLT, K/μL	2.79	2.33	2.80	0.174	0.394
MONO, %	0.543	0.332	0.262	0.074	0.230
LYM, %	35.3	31.7	34.2	0.836	0.050
NEU, %	63.3	67.1	64.7	0.886	0.067
EOS, %	0.750	0.879	0.759	0.111	0.890
BAS, %	0.042	0.023	0.035	0.016	0.592

RBC, red blood cells; HB, hemoglobin; HCT, hematocrit; MCV, mean corpuscular volume; MCH, mean corpuscular hemoglobin; MCHC, mean corpuscular hemoglobin concentration; RDW, red blood cell distribution width; PLT, platelets; MONO, monocytes; LYM, lymphocytes; NEU, neutrophil granulocytes; EOS, eosinophils granulocytes; BAS, basophil granulocytes.

Metabolic profile data are shown in Table 6. At 42 d of age, no differences were observed among the groups, except for ALT-GPT (*p* = 0.008), where the HWP group displayed a lower value compared to vitE (2.93 vs. 3.78 IU/L), and creatinine, where the GSGT group exhibited the lowest plasmatic level (*p* < 0.001). Finally, the metabolic profiles observed at 84 d were similar among dietary groups.

**Table 6:** Metabolic profile of broilers fed with different dietary treatments (vitE, control diet; GSGT, grape skin–green tea diet; HWP, hydrolyzed wood polyphenols diet).

Variables	Dietary treatments			SEM	*p*-value
	vitE	GSGT	HWP		
42 d					
Total protein, g/L	28.59	29.25	29.35	0.568	0.825
Albumins, g/L	12.06	12.25	12.15	0.207	0.925
Globulins, g/L	16.52	17.02	16.78	0.416	0.889
Albumin/globulin	0.744	0.732	0.724	0.091	0.642
Glucose, mmol/L	12.70	12.71	12.14	0.209	0.312
Triglycerides, mmol/L	0.324	0.311	0.405	0.031	0.410
Total cholesterol, mmol/L	3.87	3.86	3.83	0.068	0.952
HDL, mmol/L	2.62	2.62	2.61	0.036	0.989
LDL, mmol/L	1.18	1.18	1.16	0.038	0.977
ALT-GPT, IU/L	3.78^a^	3.17^ab^	2.93^b^	0.127	0.008
AST-GOT, IU/L	318.6	308.1	327.6	10.08	0.726
Bilirubin, μmol/L	2.38	2.38	2.42	0.061	0.970
Urea, mmol/L	0.456	0.406	0.409	0.017	0.370
CK, IU/L	9,586	9,474	10,575	609	0.724
Creatinine, μmol/L	90.51^a^	62.29^b^	89.54^a^	4.071	<0.0001
Ca, mmol/L	2.27	2.21	2.31	0.030	0.367
P, mmol/L	2.27	2.28	2.28	0.038	0.995
84 d					
Total protein, g/L	34.18	35.00	35.89	0.638	0.486
Albumins, g/L	13.89	13.76	13.92	0.149	0.915
Globulins, g/L	20.19	21.32	26.90	1.910	0.330
Albumin/globulin	0.69	0.67	0.67	0.012	0.645
Glucose, mmol/L	13.0	13.35	13.55	0.232	0.909
Triglycerides, mmol/L	0.23	0.27	0.27	0.010	0.309
Total cholesterol, mmol/L	3.00	3.15	3.10	0.048	0.606
HDL, mmol/L	2.0	2.15	2.1	0.033	0.728
LDL, mmol/L	0.91	2.41	3.86	1.110	0.534
ALT-GPT, IU/L	7.07	7.93	5.86	0.620	0.540
AST-GOT, IU/L	669	711.	693	28.2	0.733
Bilirubin, μmol/L	2.27	2.27	2.28	0.071	0.970
Urea, mmol/L	0.78	0.82	0.95	0.072	0.490
CK, IU/L	45,419	51,570	55,085	3,738	0.469
Creatinine, μmol/L	36.1	37.9	37.3	0.17	0.198
Ca, mmol/L	2.71	2.63	2.80	0.059	0.465
P, mmol/L	1.94	1.86	1.98	0.038	0.426

HDL, high-density lipoprotein; LDL, low-density lipoprotein; ALT-GPT, alanine aminotransferase–glutamate pyruvate transaminase; AST-GOT, aspartate aminotransferase–glutamate–oxaloacetate transaminase; CK, creatin kinase. ^a,b^Groups not bearing the common superscript are significantly different.

### 3.3 Immune-related gene expression

**Figure 1:**
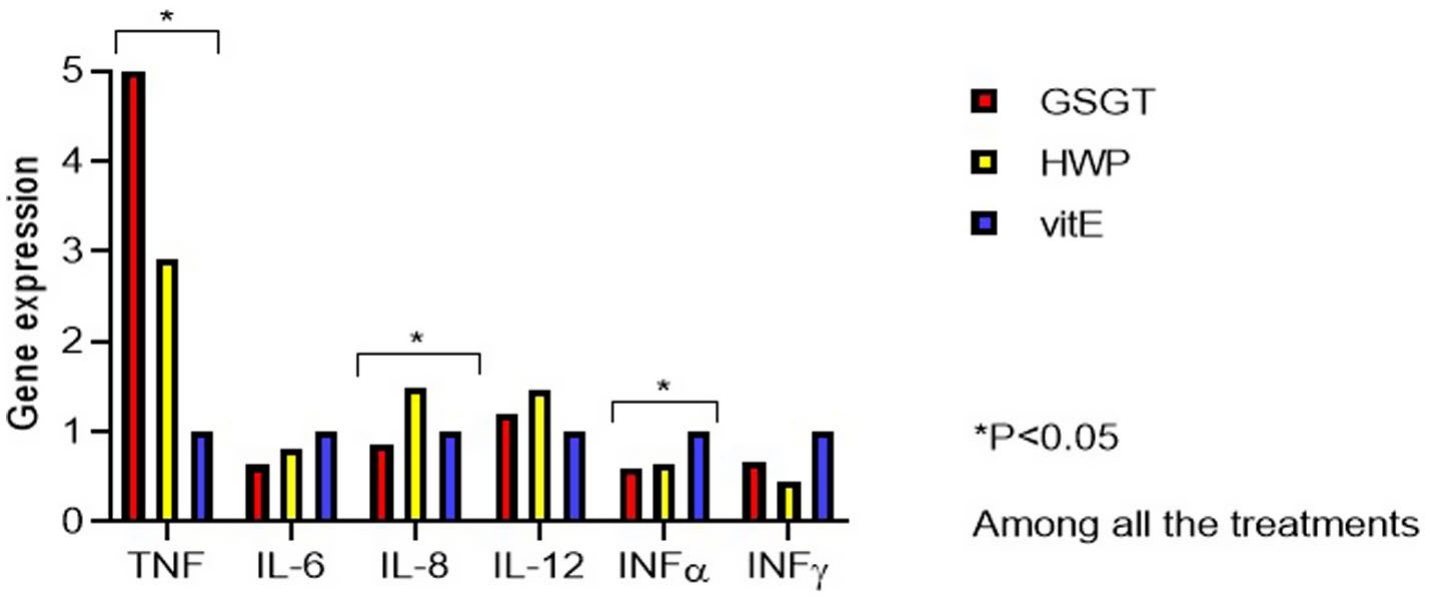
Immune-related genes expression at 42 days of the broilers fed with different dietary treatments (vitE, control diet; GSGT, grape skin–green tea diet; HWP, hydrolyzed wood polyphenols diet).

**Figure 2:**
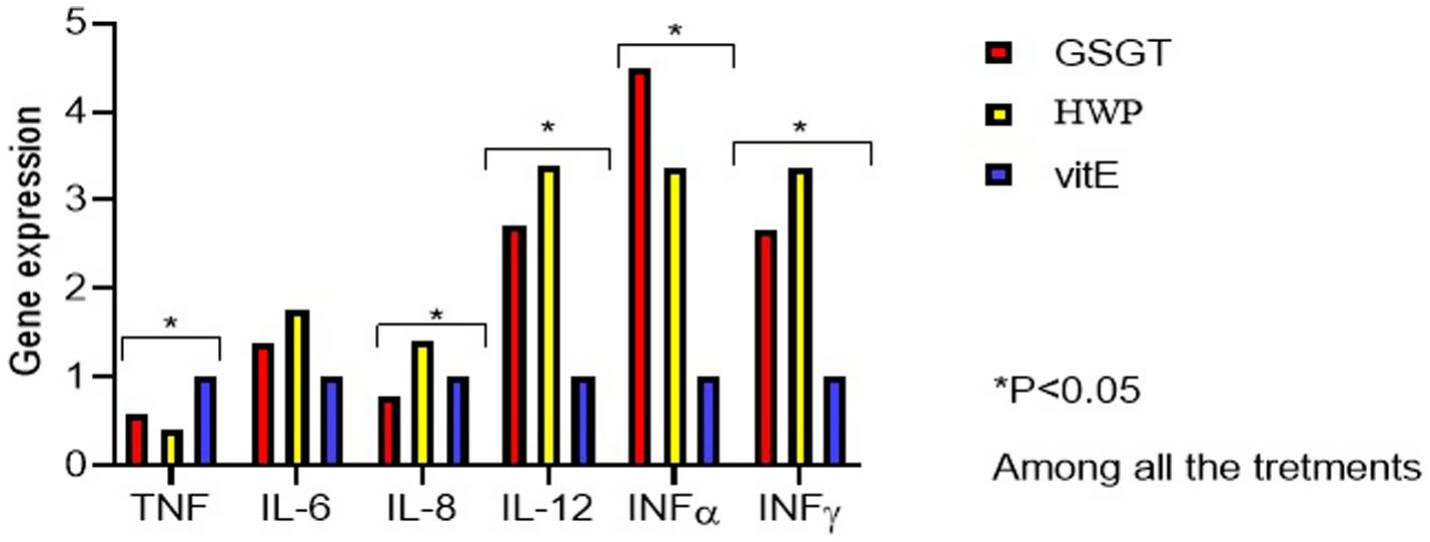
Immune-related genes expression at 84 days of the broilers fed with different dietary treatments (vitE, control diet; GSGT, grape skin–green tea diet; HWP, hydrolyzed wood polyphenols diet).

The influence of the different DT on cytokine expression as markers of the immune system response is shown in Table 7 and Figures 1, 2. In particular, both the cDNA gene expression absolute values and values expressed relative to the control group (vitE = 1) are reported.

**Table 7:** Immune-related genes expression of broilers fed with different dietary treatments (vitE, control diet; GSGT, grape skin–green tea diet; HWP, hydrolyzed wood polyphenols diet).

Variables	Dietary treatments				SEM	*p*-value
	vitE (vitE^1^)		GSGT (GSGT/vitE)	HWP (HWP/vitE)		
42 d						
TNFα	0.004^b^	(1.0)	0.018^a^ (5.03)	0.011^ab^ (2.92)	0.0034	<0.001
IL-6	0.006	(1.0)	0.004 (0.63)	0.005 (0.80)	0.0015	0.329
IL-8	0.149^ab^	(1.0)	0.126^b^ (0.85)	0.222^a^ (1.49)	0.0302	0.006
IL-12	0.0009	(1.0)	0.0010 (1.20)	0.0012 (1.46)	0.0002	0.231
INFα	0.067^a^	(1.0)	0.040^ab^ (0.60)	0.043^b^ (0.64)	0.0102	0.017
INFγ	0.006	(1.0)	0.004 (0.66)	0.003 (0.45)	0.0016	0.119
84 d						
TNFα	0.024^a^	(1.0)	0.014^ab^ (0.58)	0.010^b^ (0.40)	0.0056	0.031
IL-6	0.013	(1.0)	0.018 (1.39)	0.023 (1.75)	0.0059	0.262
IL-8	1.666^ab^	(1.0)	1.319^b^ (0.79)	2.366^a^ (1.42)	0.3008	0.003
IL-12	0.011^b^	(1.0)	0.031^ab^ (2.71)	0.039^a^ (3.38)	0.0084	0.005
INFα	0.146^b^	(1.0)	0.660^a^ (4.52)	0.489^a^ (3.35)	0.1393	0.001
INFγ	0.011^b^	(1.0)	0.030^ab^ (2.67)	0.038^a^ (3.37)	0.0083	0.007

TNFα, tumor necrosis factor alpha; IL, interleukin; IFN, interferon. ^a,b^Groups not bearing the common superscript are significantly different.

1 Values normalized by vitamin E.

At 42 d, the GSGT group compared to the vitE *group* exhibited a higher *TNFα* expression level (p < 0.001), while compared to the HWP group, a lower *IL-8* expression level was found (*p* = 0.006). Lastly, the mRNA expression of *INFα* was downregulated in the HWP group compared to the vitE group (*p* = 0.017).

*At* 84 d, the vitE group showed an increase in TNFα gene expression (*p* = 0.031) compared to the HWP group, while *IL-12* and *INFγ* were upregulated in the HWP group compared to the vitE group (*p* = 0.005 and 0.007, respectively). Additionally, the HWP group compared to the GSGT group showed higher expression of *IL-8* (*p* = 0.003). Lastly, both natural antioxidants upregulated the *INFα* expression (*p* = 0.001).

### 3.4 Carcasses and organs’ weights

Carcass and organ weights are reported in Table 8. No differences were found for carcass weight; however, concerning organ weight, the ileum of the GSGT group was heavier than that of the vitE group (+5.1 g; *p* = 0.025) at 84 d of age.

**Table 8:** Carcass and organs weights of broilers fed with different dietary treatments (vitE, control diet; GSGT, grape skin–green tea diet; HWP, hydrolyzed wood polyphenols diet).

Variables	Dietary treatments			SEM	*p*-value
	vitE	GSGT	HWP		
42 d					
Weight of, g					
Carcass	1,563	1,549	1,562	24.9	0.967
Gut	193	187	192	3.57	0.717
Proventriculus	9.20	8.97	8.89	0.25	0.864
Gizzard	31.4	34.4	33.3	0.73	0.185
Duodenum and pancreas	14.1	14.4	14.0	0.31	0.883
Jejunum	23.3	22.2	21.1	0.58	0.255
Ileum	19.9	19.1	20.2	0.52	0.662
Cecum	11.0	10.1	10.1	0.25	0.210
Heart	15.7	14.9	15.0	0.30	0.531
Liver	42.8	39.2	40.6	0.88	0.274
Colon	2.35	2.50	2.36	0.09	0.673
Spleen	1.85	2.14	2.47	0.13	0.141
Bursa of Fabricius	1.58	1.55	2.11	0.23	0.257
84 d					
Weight of, g					
Carcass	4,826	4,803	4,650	75.95	0.776
Gut	337	359	344	5.55	0.271
Proventriculus	15.1	16.9	17.2	0.41	0.656
Gizzard	55.9	61.5	55.7	1.34	0.074
Duodenum and pancreas	21.1	20.6	21.1	0.44	0.933
Jejunum	31.5	33.3	31.6	0.78	0.608
Ileum	27.8^b^	32.9^a^	29.9^ab^	0.85	0.025
Cecum	18.4	17.4	18.0	0.57	0.768
Heart	30.8	31.0	30.4	0.62	0.906
Liver	79.9	78.8	82.7	1.73	0.697
Colon	4.42	3.90	3.70	0.21	0.332
Spleen	5.43	5.17	5.30	0.14	0.756
Bursa of Fabricius	3.54	3.27	4.78	0.33	0.186
Testicles	16.2	18.1	18.0	1.09	0.737

### 3.5 Gut histomorphology

The results of the influence of DT on small and large intestine morphometry are reported in Table 9. At 42 d of age, the GSGT group compared to the vitE group exhibited a higher TMT value in the duodenum (+127 μm; *p* = 0.016) and ileum (+138 μm; *p* < 0.001). The HWP group showed the lowest TMT in the jejunum (*p* < 0.001) and a lower value in the colon than the vitE group (548 vs. 619 μM; *p* = 0.029). Regarding VH, the lowest value in the jejunum was observed for the HWP group (*p* < 0.001), while in the ileum, GSGT showed a higher measure than the vitE group (+154 μm; p < 0.001). Animals fed with HWP showed a lower VH-to-CD ratio than the control group (1.98 vs. 2.61; *p* = 0.031) and lower MMT than the GSGT group (38.6 vs. 48.7 μm; *p* = 0.026) in the colon. The groups fed with natural antioxidants showed similar measures of Circ M in the different tracts. Specifically, compared to vitE, animals fed with GSGT had higher Circ M in the ileum (+ 56 μm %; *p* = 0.009) and colon (118 μm; *p* = 0.003) while broilers fed with HWP had higher Circ M in the jejunum (+ 33 μm; *p* = 0.014). Lastly, animal fed with GSGT showed the lowest Long M in the duodenum (*p* = 0.028).

**Table 9:** Gut histomorphology of broilers fed with different dietary treatments (vitE, control diet; GSGT, grape skin–green tea diet; HWP, hydrolyzed wood polyphenols diet).

Variables	Dietary treatments			SEM	*p*-value
	vitE	GSGT	HWP		
42 d					
TMT, μm					
Duodenum	1331^b^	1458^a^	1387^ab^	19.9	0.016
Jejunum	1509^a^	1524^a^	1262^b^	25.3	<0.001
Ileum	911^b^	1049^a^	970^ab^	15.2	<0.001
Colon	619^a^	616^ab^	548^b^	13.2	0.029
Villus height, μm					
Duodenum	1,084	1,156	1,097	20.4	0.244
Jejunum	1288^a^	1299^a^	1048^b^	24.2	<0.001
Ileum	648^b^	802^a^	733^ab^	16.1	<0.001
Colon	401	380	320	13.4	0.033
Crypt depth, μm					
Duodenum	213	208	229	6.72	0.400
Jejunum	172	174	161	3.99	0.302
Ileum	178	194	178	4.83	0.265
Colon	165^b^	188^a^	189^a^	5.19	<0.001
VH/CD					
Duodenum	5.64	6.10	5.39	0.184	0.269
Jejunum	8.16	7.89	6.92	0.230	0.056
Ileum	3.97	4.43	4.63	0.154	0.190
Colon	2.61^a^	2.18^ab^	1.98^b^	0.103	0.031
MMT, μm					
Duodenum	35.9	34.1	38.6	1.08	0.207
Jejunum	34.1	36.02	38.9	1.21	0.266
Ileum	47.9	50.1	50.3	1.29	0.694
Colon	45.7^ab^	48.7^a^	38.6^b^	1.70	0.026
Circ M, μm					
Duodenum	183	184	201	6.02	0.367
Jejunum	145^b^	165^ab^	178^a^	4.58	0.014
Ileum	303^b^	359^a^	322^ab^	8.22	0.009
Colon	458^b^	576^a^	528^ab^	15.7	0.003
Long M, μm					
Duodenum	71.3^a^	53.5^b^	72.2^a^	3.29	0.028
Jejunum	58.6	53.6	62.2	2.07	0.240
Ileum	79.9	89.5	75.8	3.00	0.149
Colon	105	124	110	3.97	0.094
84 d					
TMT, μm					
Duodenum	1,527	1,412	1,410	29.1	0.166
Jejunum	1,472	1,462	1,364	20.4	0.052
Ileum	740^b^	833^a^	743^b^	17.4	0.020
Colon	551^b^	656^a^	680^a^	14.2	<0.001
Villus height, μm					
Duodenum	1,161	1,099	1,110	30.0	0.674
Jejunum	1,255	1,269	1,176	19.1	0.082
Ileum	473	537	451	16.7	0.053
Colon	288^b^	341^ab^	399^a^	11.4	<0.001
Crypt depth, μm					
Duodenum	243	246	234	6.75	0.706
Jejunum	157	150	151	3.72	0.737
Ileum	198	209	211	6.86	0.643
Colon	182	204	194	5.35	0.137
VH/CD					
Duodenum	5.21	4.89	5.15	0.208	0.801
Jejunum	8.45	9.18	8.23	0.231	0.184
Ileum	2.99	3.02	2.47	0.147	0.218
Colon	1.65^b^	1.86a^b^	2.34^a^	0.097	0.007
MMT, μm					
Duodenum	44.0^a^	43.9^ab^	38.3^b^	1.90	0.044
Jejunum	41.1^a^	31.5^b^	35.8^ab^	1.20	0.002
Ileum	57.7^ab^	66.8^a^	48.2^b^	2.06	<0.001
Colon	61.4	51.9	53.6	2.46	0.322
Circ M, μm					
Duodenum	231^a^	238^a^	192^b^	5.42	<0.001
Jejunum	191^a^	156^b^	187^a^	4.42	<0.001
Ileum	494	481	455	17.2	0.320
Colon	888	873	783	33.3	0.310
Long M, μm					
Duodenum	99.6^a^	88.9^a^	63.5^b^	3.29	<0.001
Jejunum	73.9^a^	52.7^b^	60.4^b^	2.45	<0.001
Ileum	142.4^a^	128.7^ab^	110.8^b^	5.00	0.024
Colon	206.2^ab^	228.6^a^	170.3^b^	7.99	0.003

TMT, tunica mucosa thickness; VH, villus height. CD, crypt depth. MMT, lamina muscolaris mucosae thickness. CircM, thickness of the inner circular; Long M, longitudinal layers of the tunica muscolaris. ^a,b^Groups not bearing the common superscript are significantly different.

At 84 days of age, no differences were found for CD. Natural antioxidants led to the highest TMT in the colon (*p* < 0.001), and animals fed with GSGT showed the highest TMT in the ileum (*p* = 0.020). Additionally, HWP resulted in higher VH than vitE (+111 μm; *p* < 0.001) and the highest VH/CD value (*p* = 0.007) in the colon. Concerning Circ M, the lowest value was observed for the HWP group in the duodenum (−42 μm, on average; p < 0.001) and for the GSGT group in the jejunum (−33 μm, on average; p < 0.001). Lastly, concerning Long M, HWP showed the lowest thickness in the duodenum (p < 0.001) and, compared to vitE, a lower value in the ileum (110.8 vs. 142.4 μm; *p* = 0.024), while compared to GSGT, a lower thickness in the colon (170.3 vs. 228.6 μm; *p* = 0.003). Additionally, both natural antioxidants decreased the Long M thickness in the jejunum (p < 0.001).

### 3.6 Meat quality

The effect of DT on meat chemical composition, technological, and quality traits of chicken meat is reported in Table 10. The only difference was observed for protein at 42 days of age, with the GSGT-fed groups showing a higher protein content in meat compared to those fed with HWP (22.6 vs. 22.1%; *p* = 0.024).

**Table 10:** Meat quality, color, and pH values of breast muscle (*pectoralis major*) of broilers fed with different dietary treatments (vitE, control diet; GSGT, grape skin–green tea diet; HWP, hydrolyzed wood polyphenols diet).

Variables	Dietary treatments			SEM	*p*-value
	VitE	GSGT	HWP		
42 d					
Moisture, %	69.4	69.4	69.5	0.06	0.557
Fat, %	2.93	2.74	2.88	0.07	0.527
Protein, %	22.2^ab^	22.6^a^	22.1^b^	0.07	0.024
pH	5.80	5.76	5.78	0.01	0.304
Lightness, L	53.0	53.4	53.3	0.24	0.769
Redness, a	2.68	2.69	2.47	0.08	0.421
Yellowness, b	7.62	8.08	7.40	0.12	0.052
Shear force, N/g	33.5	31.0	31.9	0.67	0.297
Purge loss, %	23.9	21.8	23.4	0.46	0.172
84 d					
Moisture, %	68.4	67.9	66.9	0.92	0.845
Fat, %	4.61	4.60	4.66	0.150	0.982
Protein, %	22.7	22.5	22.5	0.133	0.799
pH	5.75	5.72	5.71	0.010	0.428
Lightness, L	55.5	56.3	56.0	0.311	0.519
Redness, a	2.54	2.31	2.31	0.093	0.501
Yellowness, b	7.11	7.38	7.18	0.180	0.808
Shear force, N/g	51.0	49.1	51.7	1.30	0.668
Purge loss, %	46.5	48.2	47.8	0.99	0.756

^a,b^Groups not bearing the common superscript are significantly different.

### 3.7 Meat cuts weight

Table 11 shows the results of the different DT on the weight of meat cuts, and no differences were found throughout the trial.

**Table 11:** Meats cut weights of broilers fed with different dietary treatments (vitE, control diet; GSGT, grape skin–green tea diet; HWP, hydrolyzed wood polyphenols diet).

Variables	Dietary treatments			SEM	*P*-value
	VitE	GSGT	HWP		
42 d					
Whole chicken, g	1,539	1,529	1,539	25.0	0.982
Feet, g	97.1	93.7	99.8	1.76	0.371
Wings, g	151	155	154	2.42	0.712
Leg quarter, g	439	447	441	7.49	0.920
Whole breast, g	504	477	496	9.50	0.485
84 d					
Whole chicken, g	4,804	4,920	4,789	84.4	0.666
Feet, g	199	200	194	2.96	0.509
Wings, g	451	458	439	6.87	0.537
Leg quarter, g	1,420	1,450	1,359	26.1	0.356
Whole breast, g	1,610	1,685	1,687	35.8	0.607

### 3.8 Survival rate

Survival rate was not affected by DT in the two considered periods: 0–42 and 43–84 days of age. Average values of 95.2, 95.2, and 91.0% and of 70.5, 79.6, and 75.7% were observed between 0 and 42 days of age and 43 and 84 days of age for vitE, GSGT, and HWP, respectively. The EC survival rate between 0 and 84 days of age was similar among the groups, being 67.2, 75.7, and 69.3% for vitE, GSGT, and HWP, respectively.

### 3.9 European poultry efficiency factor (EPEF)

No statistical differences were observed among the EPEF of the groups. Regarding the evaluation of EPEF in the period between 0 and 42 d of age, the HWP group showed the lowest value compared to GSGT and vitE (293.0 vs. 310.4 and 309.6, respectively; *p* = 0.484), while in the period between 43 and 84 days, the EPEF of the GSGT group was slightly higher than those of vitE and HWP (283.3 vs. 223.5 and 245.1; *p* = 0.456). In the period between 0 and 84 d of age, the GSGT group showed higher EPEF than vitE and HWP (251.4 vs. 206.1 and 214.9; *p* = 0.454).

## 4 Discussion

Synthetic vitamin E, a widely used antioxidant in poultry farming, may be replaced by PFA, particularly by natural polyphenolic compounds (2). This substitution is encouraged by the European Union in organic livestock production. Two PFAs were tested in the present study as substitutes for vitamin E in poultry diets at doses calculated based on their *in vitro* measured antioxidant capacity to obtain the same dietary oxidative status. To the best of our knowledge, this approach has never been applied in other animal antioxidant studies, where the doses of PFAs were generally based on recommendations by the producers or retrieved from the literature. Furthermore, the effects of replacing synthetic vitamin E with natural alternatives were tested in the present study on broilers raised under organic-like conditions for a period typical of the conventional rearing cycle (42 d), allowing for comparison with existing literature, and for an interval of 84 days, compliant with the organic rearing cycle [minimum 81 d; (40)].

Contrasting results on the effects of PFAs on animal performance have been reported in the literature, mainly due to differences in dosages and botanical compositions of the products tested across studies. Overall, the natural antioxidants tested in the present trial did not affect production performance or BW, showing similar results to synthetic vitamin E (41, 42). This is consistent with the results reported by other authors evaluating grape by-products (15 to 60 g/kg) in comparison to α-tocopherol acetate (200 mg/kg) in Cobb strain broilers, as well as grape stem extracts against a negative control diet in Ross-308 broilers (41, 42). Moreover, despite a lower FCR during the initial 21 days, mainly due to the organic-like rearing conditions adopted, our findings are consistent with those obtained using similar PFAs such as Kombucha tea and green tea in Ross 308 broilers at 42 days of rearing (43, 44). Conversely, when raw and fermented grape pomace were tested at 15 g/kg against both negative and positive control diets, the group receiving synthetic antioxidants (0.25 g/kg) exhibited a lower FCR and higher BW (45). In another study, the dietary inclusion of green tea at 0.750 g/kg enhanced BW in comparison to the negative control diet, whereas 0.500 g/kg increased feed intake and FCR (46).

The absence of differences among DT clearly indicates that the study’s approach—based on equating groups for antioxidant activity—is effective and allows a meaningful comparison between natural and synthetic molecules. This indicates how PFAs, when correctly dosed based on their antioxidant activity, do not adversely affect the animals’ growth performance compared to synthetic antioxidants. Furthermore, despite the limitations related to the low number of pen replicates in the present study, these results are important from a practical perspective since both tested products appear to be suitable substitutes for vitamin E in diets used in both conventional and organic farming systems.

Vitamins E and A are lipophilic compounds that share common uptake pathways, relying on membrane transporters and lipid-binding proteins such as SR-BI (Scavenger Receptor Class B Type I), NPC1L1, and CD36, which facilitate the selective absorption of these micronutrients into enterocytes. Variation in vitamin E levels may influence vitamin A intestinal absorption, consequently affecting the bioavailability of other lipophilic molecules (e.g., lutein) due to competitive interactions during intestinal absorption (47, 48). As expected, the plasma level of tocopherol in the vitE group was higher compared to the other groups. Interestingly, higher levels of vitamin A were observed in the HWP group. These findings suggest that both plasmatic and hepatic vitamin A concentrations are influenced not only by the digestibility and absorption of dietary carotenoids (49) but also by the presence of plant-derived polyphenols, which can modulate carotenoids’ bioavailability either positively or negatively (50). More specifically, hesperetin, hesperidin, and naringenin are reported to enhance the intestinal absorption of *β*-carotene (50), while catechins, which represent the main polyphenols in green tea, depress carotenoid absorption (51). Phenolic acids and tannins included in HWP are reported to interfere as antinutritional factors with protein digestion and with digestive enzymes such as *α*-amylase, α-glucosidase, pancreatic lipase, pepsin, and trypsin (52). This could explain the lower protein content and yellowness observed in the meat of HWP, despite the higher plasmatic vitamin A levels found at 42 days of age. Despite the differences in vitamin levels, the final antioxidant effect did not change with DT because of the inclusion of the natural alternatives employed at specific dosages. Therefore, both GSGT and HWP can be considered valid antioxidant alternatives to synthetic vitamin E when properly dosed. This finding underscores the importance of balancing animal diets for their antioxidant capacity, implying a preliminary laboratory measurement of the antioxidant capacity of the additives to be utilized. Specifically, since it is well known that the reference antioxidant is vitamin E, the doses of the natural antioxidants were successfully adjusted in the present trial based on that vitamin, through the ABTS test, which is based on Trolox, the water-soluble analog of vitamin E (23).

The potential of PFA to affect blood cell count and the metabolic profile of animals depends on both the type of active principle they contain and the inclusion doses (2, 53, 54). In our study, neither the type of PFA used nor the inclusion dose resulted in any significant difference compared to the control for blood parameters. This is consistent with a previous study that included 1.5% of green tea in the basal diet of broilers, either alone or in combination with dexamethasone injection. The effects were compared to those of vitamin E and a negative control in animals reared up to 42 days of age (55). Concerning the metabolic profiles, at the dosage tested in our study, no significant differences were observed between groups in lipid metabolism parameters or total protein levels at 42 days. Although HWP animals slaughtered at day 42 showed a numerically higher triglyceride content, this difference did not reach statistical significance, likely due to the relatively high variability of the data and the limited number of replicates. This is in accordance with other studies, which report no differences in cholesterol levels, lipid profile, and triglycerides when testing olive oil by-products (from 0 to 30 mg/kg with intervals of 7.5 mg/kg) as a substitute for vitamin E (ranging from 0 to 40 mg/kg with intervals of 10 mg/kg) on Cobb 500 broilers up to 39 days of age (56). However, some studies have reported that PFA can express hypocholesterolemic properties when compared to both vitamin E-positive and vitamin E-negative controls (2). In particular, the inclusion of grape pomace and green tea extract into the diet can lead to a reduction in total cholesterol levels while simultaneously increasing HDL levels when compared to negative control diets (43). Additionally, other authors tested different inclusion levels of grape seed extract (doubling the doses in the range from 125 to 2000 ppm) in Ross 308 broilers up to 42 days of age, reporting lower levels of cholesterol and LDL compared to the control animals fed synthetic antioxidants (57). An increase of triglycerides, cholesterol, and LDL was also observed when broilers were fed different concentrations of green tea (250 to 750 g/kg) up to 6 weeks of age in comparison to a negative control diet (46). Furthermore, in another study on Ross-308 broilers raised until 42 days of age, rosemary by-products compared to vitamin E (50 mg/kg and 200 mg/kg) decreased cholesterol levels, but this effect was associated with an increase in liver enzymes and creatinine (58). Existing literature extensively reports a decrease in liver enzymes in animals fed with PFA compared to those receiving vitamin E and/or C or with a negative diet (2). However, this reduction is dose-dependent, as higher doses may lead to adverse effects, resulting in an increase of both alanine aminotransferase (ALT) and aspartate aminotransferase (AST) (46). In our study, HWP animals showed a reduction in ALT. As reported in the literature, polyphenols are absorbed by the small intestine (about 5–10% of the total ingested), metabolized by enterocytes and hepatocytes, and finally excreted via the kidneys in urine (59). The lower ALT values in HWP animals could relate, along with a beneficial antioxidant effect exerted by hydrolyzed polyphenols on the hepatocytes, to the simpler structure of the hydrolyzed form, allowing for easier metabolization and lower metabolic load on hepatocytes compared to non-hydrolyzed polyphenols. Lastly, another important metabolic parameter is creatinine, which is a marker of renal function, and its concentration in the blood is directly proportional to muscle mass and depends on factors such as age, physical activity, and diet (60). In our study, GSGT led to the lowest levels of creatinine at 42 days of age. Since there were no differences in BW and carcass weight of the animals, the lower creatinine values could indicate a possible beneficial effect of the grape and green tea mixture relating to their antioxidant compounds content; in particular, a beneficial effect of green tea on the kidney function of hens has been reported (61). However, for this parameter, it is crucial to consider the tested dosage, since when green tea was included alone (from 250 to 750 g/kg) in comparison to the negative diet, an increase in creatinine was observed.

Throughout the duration of our study, no differences were observed in hematological analyses, particularly regarding the white blood cell population and blood globulins. However, the overall results on cytokine gene expression showed an upregulation during the second phase of the trial, probably due to multiple factors, including the increased body weight of the broiler strain 308—which is conventionally reared in shorter production cycles than 80 days—as well as changes in the histomorphological traits of the small and large intestine observed during the trial (62).

The PBMC are key components of the peripheral immune system, easily accessible and highly responsive to immunological stimuli. Consequently, cytokine expression by these cells serves as a direct indication of the immunomodulatory effect of the nutrients tested. The *TNFα* expression was higher in GSGT than in vitE at 42d, and it is known that this molecule regulates the production of other inflammatory mediators (63). However, no clinical or analytical indicators of inflammation were found in this interval. On the other hand, at the 84 d interval, an upregulation of IL-12, INFα, and INF*γ*, along with a downregulation of TNFα expression was found, particularly in the HWP group. The expression of inflammatory cytokines, including IL-12, is strongly related to the expression of INFα and γ and may be upregulated during infections and chronic diseases (64, 65). In the latter case, many polyphenols have been demonstrated to reduce TNFα, IL-1, and IL-6 expressions in both *in vitro* and *in vivo* trials (66, 67). Despite only a few studies having elucidated the functions of IL-12, it is generally considered pro-inflammatory and prostimulatory (68), increasing interferon expression (69). On the other side, it has been demonstrated that broilers supplemented with postbiotics showed a higher baseline expression of interferons (INFs) in the thymus and spleen, which participate in the maturation of Th1 cells, as well as in leukocyte attraction and the regulation of B-cell immunoglobulin production (70, 71). Thus, it was hypothesized that these additives can keep the innate immune system of supplemented animals in a “standby” or “alert mode,” where it is prepared and ready to respond to immune stimuli faster than in control animals (72). In our study, the same higher INFs baseline was observed in the presence of natural antioxidants, particularly in the HWP group, leading to a similar hypothesis concerning the “alert” mode. Despite the weakness of the statistical analysis for this parameter, due to the low number of repetitions, considering that we did not quantify macrophage functionality and pro-inflammatory cytokines in the blood, and given the absence of differences in antioxidant traits, blood cell counts, and metabolic profile, animal performance and survival rate, along with a higher EPEF in the natural antioxidant groups, the hypothesis of the “alert” mode seems the most probable.

The impact of PFAs on carcass and organ weight has been debated in the literature, with evidence suggesting that both the type and dosage of PFA can influence organ and carcass weight, often leading to contradictory results (3). A study reported that including green tea extract can affect carcass and organ weight due to its positive effect on growth performance (44). In our study, no differences were observed in carcass and organ weight, except for the ileum. It should be noted that Ross 308 carcass yields at 42d are usually higher compared to our results, reaching 70–73%. However, this result can be partially explained, as previously mentioned, by the organic-like conditions in which our animals were raised. As reviewed by Pitino et al. (3), several studies have focused not only on evaluating carcass and organ yields but also on meat quality, an aspect that has become increasingly important to consumers. In our study, the GSGT animals had a higher protein content in meat than those fed HWP. This could be due to the greater ability of animals in this group to absorb macronutrients, as GSGT positively affected the Jejunum VH and TMT. In fact, the mucosal and muscular layers of the intestines play crucial roles in digestive tract morpho-physiology and diagnosing digestive system diseases. The mucosal layer produces mucus, facilitating the passage of chyme and protecting the gastrointestinal tract from digestive enzymes (73). Meanwhile, the muscular layer contributes to peristalsis and propels the chyme along the intestine. Consequently, an increase in the thickness of these intestinal layers (muscle and mucosa) is associated with improved intestinal health (74). At 42 days, the duodenal and ileal tunica mucosa, and at 84 days, the colic tunica mucosa, were thicker in GSGT broilers than in those treated with vitE. Conversely, the HWP diet led to a thinning of the jejunal tunica mucosa at 42 d compared to other groups. Although TMT correlates with the length of intestinal villi, which are the primary site of nutrient absorption (75), VH was notably greater at 42 days only in the jejunum and ileum of the GSGT groups DT, showing values similar to vitE and HWP, respectively, in the two intestinal tracts. Another parameter closely linked to TMT and, therefore, intestinal health and animal response, is CD (74). In healthy animals, greater CD is related to the migration of specialized cells towards the villi, influenced by the shortening and decreased functionality of these structures due to multifactorial causes, like the ingestion of litter particles to compensate for a low-fiber diet and the subsequent abrasive effect of these fibers on the intestinal epithelium. In our study, we did not find differences in CD for the entire trial. Furthermore, it’s important to consider the relationship of the VH/CD, which is used as a nutritional and health indicator to estimate digestive processes and nutrient absorption in the gut (76). Thus, the greatest capacity for digestion and absorption occurs when this ratio increases (77, 78). In our study, for the whole duration of the test, differences were found only for the colon, while no differences were found in the small intestine. Under conventional broiler rearing conditions (42-day cycle), a normal survival rate for broiler Ross 308 is considered around 97% (16), and the EPEF value should be higher than 300 to have a profitable rearing cycle (79). In the present study, no differences among the DT were found for these two parameters. However, the low number of repetitions could have masked several results, representing, in fact, the main limitation of the work. In general, the lower survival rate found at 42 d may be attributed to the organic-like condition in which the animals were reared. Particularly, the lowest survival rate was found in HPW at 42 d, while in the long rearing cycle, the GSGT group had the highest survival rate, resulting in a higher EPEF value for the same group of animals. Phytogenic additives have been shown to increase EPEF by 31 points in a recent experiment (80). Additionally, the GSGT group was the only one to reach values higher than 250 when considering the organic rearing cycle. This suggests that natural antioxidants can be recommended in long rearing cycles conducted according to organic farming guidelines.

## 5 Conclusion

This study demonstrates that HWP and GSGT can serve as viable alternatives to synthetic vitamin E, provided they are tested for *in vitro* antioxidant activity and used at doses that allow for balancing animal diets in terms of antioxidant capacity. These alternatives do not adversely affect growth performance, health status, carcass weight, meat quality, histomorphology, or survival rate. Moreover, they exhibit a positive impact on the metabolic profile and health status of the animals and efficient production, albeit with some specific variability.

## Data Availability

The data presented in this study are available free of charge for any user upon reasonable request from the corresponding authors.
