# Supplementary material for: Effects of antioxidant capacity-based dietary replacement of vitamin E with commercial products containing grape skin–green tea extracts or hydrolyzed wood polyphenols on poultry performance, metabolism, immune-related gene expression, and meat quality
Source: Front Vet Sci. 2025 Jul 7;12:1608147. doi: 10.3389/fvets.2025.1608147 (PMC12278287; doi:10.3389/fvets.2025.1608147)
Supplement: Supplementary file 1 [file Table_1.docx]

Supplementary Material

**Table Supplementary 1**. Sequences of PCR primers used for Peripheral Blood mononuclear cells (PBMC) genes expression.

| Target gene | Primer sequence | | | | References^1^ |
| --- | --- | --- | --- | --- | --- |
|  | Forward |  | | Reverse |  |
| IL6 | 5′- GCTCGCCGGCTTCGA-3′ | | 5′- GGTAGGTCTGAAAGGCGAACAG-3′ | | (Connerton, P.L., 2018) |
| IL8 | 5’- GCCCTCCTCCTGGTTTCA-3’ | | 5’- CGCAGCTCATTCCCCATCT-3’ | | (Peng L., 2020) |
| IL12 | 5’- AGATGCTGGCAACTACACCTG-3’ | | 5’- CATTTGCCCATTGGAGTCTAC-3’ | | (Hong Y., 2020) |
| IFNα | 5′- CCAGCACCTCGAGCAAT-3′ | | 5′- GGCGCTGTAATCGTTGTCT-3′ | | (Y. P. Li, 2007) |
| IFNγ | 5’- ATCATACTGAGCCAGATTGTTTCG-3’ | | 5’-TCTTTCACCTTCTTCACGCCAT-3’ | | (Saeed, 2018) |
| TNF-α | 5′- GCCCTTCCTGTAACCAGATG -3′ | | 5′- ACACGACAGCCAAGTCAACG -3′ | | (Wang Y, 2017) |
| ACTB | 5’- TGCTGTGTTCCCATCTATCG-3’ | | 5’- TTGGTGACAATACCGTGTTCA-3’ | | (Hu, 2020) |
| GAPDH | 5’- TGCTGCCCAGAACATCATCC-3’ | | 5’- ACGGCAGGTCAGGTCAACAA-3’ | | (Hong Y., 2020) |
| HPRT-1 | 5’- AAGTGGCCAGTTTGTTGGTC-3’ | | 5’- GTAGTCGAGGGCGTATCCAA-3’ | | (Boo SY, 2020) |

^1^List of references

Boo SY, Tan SW, Alitheen NB, Ho CL, Omar AR, Yeap SK. Identification of Reference Genes in Chicken Intraepithelial Lymphocyte Natural Killer Cells Infected with Very-virulent Infectious Bursal Disease Virus. Sci Rep. 2020 May 22;10(1):8561. doi: 10.1038/s41598-020-65474-3.

Connerton, P.L., Richards, P.J., Lafontaine, G.M. et al. The effect of the timing of exposure to Campylobacter jejuni on the gut microbiome and inflammatory responses of broiler chickens. Microbiome 6, 88 (2018). https://doi.org/10.1186/s40168-018-0477-5

Peng, L., Scheenstra, M.R., van Harten, R.M. et al. The immunomodulatory effect of cathelicidin-B1 on chicken macrophages. Vet Res 51, 122 (2020). https://doi.org/10.1186/s13567-020-00849-y

Hong Y, Lee J, Vu TH, Lee S, Lillehoj HS, Hong YH. Chicken avian β-defensin 8 modulates immune response via the mitogen-activated protein kinase signaling pathways in a chicken macrophage cell line. Poult Sci. 2020 Sep;99(9):4174-4182. doi: 10.1016/j.psj.2020.05.027.

Li, Y., Handberg, K., Juul-Madsen, H. et al. Transcriptional profiles of chicken embryo cell cultures following infection with infectious bursal disease virus. Arch Virol 152, 463–478 (2007). <https://doi.org/10.1007/s00705-006-0878-9>

Saeed, M.; Yatao, X.; Hassan, F.-u.; Arain, M.A.; Abd El-Hack, M.E.; Noreldin, A.E.; Sun, C. Influence of Graded Levels of l-Theanine Dietary Supplementation on Growth Performance, Carcass Traits, Meat Quality, Organs Histomorphometry, Blood Chemistry and Immune Response of Broiler Chickens. Int. J. Mol. Sci. 2018, 19, 462. https://doi.org/10.3390/ijms19020462

Hu, H.; Chen, L.; Dai, S.; Li, J.; Bai, X. Effect of Glutamine on Antioxidant Capacity and Lipid Peroxidation in the Breast Muscle of Heat-stressed Broilers via Antioxidant Genes and HSP70 Pathway. Animals 2020, 10, 404. https://doi.org/10.3390/ani10030404

**Table Supplementary 2.** Growth performance, feed and water consumptions of broilers fed with different dietary treatments (vitE= control diet; GSGT= grape skin-green tea diet; HWP= hydrolysed wood polyphenols diet)

| Variables | Dietary Treatments | | | SEM | *P*-value |
| --- | --- | --- | --- | --- | --- |
|  | vitE | GSGT | HWP |  |  |
| 0 to 7 d of age |  |  |  |  |  |
| FC, g | 134.1 | 132.8 | 132.2 | 1.22 | 0.880 |
| WC, L | 0.54 | 0.50 | 0.39 | 0.040 | 0.446 |
| BWG, g | 139.1 | 132.3 | 134.7 | 0.030 | 0.101 |
| FCR | 0.75 | 0.76 | 0.74 | 0.013 | 0.513 |
| W:F | 3.93 | 3.11 | 2.93 | 0.323 | 0.480 |
| 0 to 14 d of age |  |  |  |  |  |
| FC, g | 488.1 | 472.1 | 490.2 | 6.09 | 0.474 |
| WC, L | 1.347 | 1.405 | 1.241 | 0.10 | 0.791 |
| BWG, g | 417.6 | 397.3 | 419.4 | 5.51 | 0.319 |
| FCR | 1.067 | 1.062 | 1.056 | 0.01 | 0.780 |
| W:F | 2.76 | 2.81 | 2.54 | 0.133 | 0.723 |
| 0 to 21 d of age |  |  |  |  |  |
| FC, g | 1130 | 1131 | 1166 | 13.1 | 0.497 |
| WC, L | 3.09 | 2.97 | 3.15 | 0.093 | 0.568 |
| BWG, g | 831.4 | 806.8 | 845.8 | 8.39 | 0.280 |
| FCR | 1.30 | 1.33 | 1.33 | 0.012 | 0.444 |
| W:F | 2.73 | 2.56 | 2.71 | 0.092 | 0.751 |
| 0 to 28 d of age |  |  |  |  |  |
| FC, g | 2012 | 2023 | 2062 | 14.4 | 0.379 |
| WC, L | 5.24 | 5.02 | 5.25 | 0.118 | 0.567 |
| BWG, g | 1335 | 1334 | 1365 | 7.88 | 0.322 |
| FCR | 1.46 | 1.50 | 1.51 | 0.007 | 0.358 |
| W:F | 2.61 | 2.44 | 2.55 | 0.059 | 0.645 |
| 0 to 35 d of age |  |  |  |  |  |
| FC, g | 3113 | 3134 | 3129 | 16.3 | 0.890 |
| WC, L | 7.84 | 7.36 | 7.54 | 0.178 | 0.514 |
| BWG, g | 1948 | 1957 | 1939 | 20.1 | 0.932 |
| FCR | 1.57 | 1.59 | 1.65 | 0.017 | 0.142 |
| W:F | 2.52 | 2.33 | 2.41 | 0.057 | 0.396 |
| 0 to 42 d of age |  |  |  |  |  |
| FC, g | 4424 | 4450 | 4399 | 34.3 | 0.869 |
| WC, L | 11.7 | 11.2 | 12.2 | 0.24 | 0.244 |
| BWG, g | 2437 | 2446 | 2409 | 26.5 | 0.621 |
| FCR | 1.71 | 1.75 | 1.76 | 0.02 | 0.602 |
| W:F | 2.58^ab^ | 2.46^b^ | 2.67^a^ | 0.04 | 0.042 |
| WE | 0.224 | 0.235 | 0.214 | 0.004 | 0.104 |
| WCR | 4.45 | 4.26 | 4.66 | 0.078 | 0.093 |
| 0 to 63 d of age |  |  |  |  |  |
| FC, g | 9192 | 9381 | 9011 | 134.2 | 0.595 |
| WC, L | 28.6 | 27.3 | 28.6 | 1.13 | 0.870 |
| BWG, g | 5024 | 4804 | 4665 | 94.0 | 0.370 |
| FCR | 2.01 | 1.94 | 2.05 | 0.035 | 0.626 |
| W:F | 2.94 | 2.73 | 2.96 | 0.009 | 0.643 |
| 0 to 84 d of age |  |  |  |  |  |
| FC, g | 14725 | 14569 | 14449 | 337 | 0.995 |
| WC, L | 59.5 | 59.0 | 58.8 | 2.85 | 0.995 |
| BWG, g | 6117 | 6325 | 6107 | 114 | 0.567 |
| FCR | 2.25 | 2.15 | 2.24 | 0.05 | 0.754 |
| W:F | 3.67 | 3.70 | 3.73 | 0.16 | 0.990 |
| WE | 0.130 | 0.127 | 0.127 | 0.008 | 0.974 |
| WCR | 7.79 | 8.03 | 7.87 | 0.327 | 0.964 |

Abbreviations: FC, feed consumption; WC, water consumption; BWG, body weight gain; FCR, feed conversion ratio; W:F, water to feed ratio; WCR, water conversion ratio.
